# Efficacy of *Metarhizium anisopliae*, Isolate ICIPE 7, against *Anopheles arabiensis*, *Glossina fuscipes*, and *Rhipicephalus* spp.

**DOI:** 10.3390/insects15060449

**Published:** 2024-06-13

**Authors:** Fedinand Ong’wen, Margaret Mendi Njoroge, Ulrike Fillinger, Heike Lutermann, Tullu Bukhari

**Affiliations:** 1 Human Health Theme, International Centre of Insect Physiology and Ecology, Nairobi P.O. Box 30772-00100, Kenya; mnjoroge@icipe.org (M.M.N.); ufillinger@gmail.com (U.F.); tbukhari@icipe.org (T.B.); 2 Department of Zoology and Entomology, Faculty of Natural & Agricultural Sciences, University of Pretoria, Private Bag x 20, Hatfield 0028, South Africa; hlutermann@zoology.up.ac.za

**Keywords:** biorational, integrated vector control, *Metarhizium anisopliae*, ICIPE 7, ICIPE 30, malaria mosquitoes, tsetse flies, ticks

## Abstract

**Simple Summary:**

Arthropods are a large and diverse group of animals, some of which are pests, while others transmit human and livestock diseases as they bite them to feed on blood. Chemical-based vector and pest control for decades has made many arthropods resistant to the chemicals used. So, there is a need to investigate other control agents, such as the biological, arthropod-killing fungus *Metarhizium anisopliae*, for the ability to infect and kill human and livestock disease-transmitting arthropods. In this study, we tested if the fungus *M. anisopliae* infects and kills mosquitoes, tsetse flies, and ticks. This was performed by finding out how long mosquitoes lived after being infected with fungus spores, as well as the amount of fungus spores suitable to lead to their death. The same optimal concentration of fungus that killed mosquitoes also successfully killed tsetse flies and ticks. We recommend that the fungus be formulated in a suitable concentration of 10^9^ spores/mL and its ability to kill mosquitoes, tsetse flies, and ticks be confirmed in the field. This would lead to the development of a fungal formulation that can be sprayed on livestock such as cattle, for the exposure of multiple blood-feeding and disease-transmitting arthropods at the same time, to impact their survival.

**Abstract:**

Arthropod vectors are responsible for a multitude of human and animal diseases affecting poor communities in sub-Saharan Africa. Their control still relies on chemical agents, despite growing evidence of insecticide resistance and environmental health concerns. Biorational agents, such as the entomopathogenic fungus *Metarhizium anisopliae*, might be an alternative for vector control. Recently, the *M. anisopliae* isolate ICIPE 7 has been developed into a commercial product in Kenya for control of ticks on cattle. We were interested in assessing the potential of controlling not only ticks but also disease-transmitting mosquitoes and tsetse flies using cattle as blood hosts, with the aim of developing a product for integrated vector management. Laboratory bioassays were carried out with *M. anisopliae*, isolate ICIPE 7 and isolate ICIPE 30, to compare efficacy against laboratory-reared *Anopheles arabiensis*. ICIPE 7 was further tested against wild *Glossina fuscipes* and *Rhipicephalus* spp. Dose–response tests were implemented, period of mosquito exposure was evaluated for effects on time to death, and the number of spores attached to exposed vectors was assessed. Exposure to 10^9^ spores/mL of ICIPE 7 for 10 min resulted in a similar mortality of *An. arabiensis* as exposure to ICIPE 30, albeit at a slower rate (12 vs. 8 days). The same ICIPE 7 concentration also resulted in mortalities of tsetse flies (LT_50_: 16 days), tick nymphs (LT_50_: 11 days), and adult ticks (LT_50_: 20 days). Mosquito mortality was dose-dependent, with decreasing LT_50_ of 8 days at a concentration of 10^6^ spores/mL to 6 days at 10^10^ spores/mL. Exposure period did not modulate the outcome, 1 min of exposure still resulted in mortality, and spore attachment to vectors was dose-dependent. The laboratory bioassays confirmed that ICIPE 7 has the potential to infect and cause mortality to the three exposed arthropods, though at slower rate, thus requiring further validation under field conditions.

## 1. Introduction

Vector-borne diseases account for 17% of the global burden of all infectious diseases, with the heaviest burden in Africa, Asia, and South America [1,2]. These diseases are a major cause of morbidity and mortality in both humans and livestock in developing countries [1,2]. Challenges such as over-reliance on one or a few control agents, insecticide resistance, and climate change, as well as uncoordinated and inconsistent disease surveillance, affect the success of current interventions [3,4,5,6,7]. These challenges, together with insecticide dependence for both human and livestock disease vector control and effects on non-target organisms as well as the environment [8,9], have led to better control approaches being sought. Also, limited resources in vector control programs and the requirement as per current WHO frameworks to integrate vector control interventions and work across sectors [10] have brought in the need to search for alternative, effective, and safe vector control agents. Based on this need, testing and developing a potential agent that is able to infect and kill several human and livestock disease vectors at once is a promising venture for a One Health vector control approach [11].

Malaria is among the most important vector-borne diseases in humans. In 2021, nearly half of the human population was at risk of getting infected with the disease [12]. Though insecticide-based vector control has played an important part in the reduction of malaria cases globally, there has been an increase in the disease incidences since 2016 [12]. The increase is partly attributed to insecticide resistance [13,14,15] and the change in behavior of primary malaria vectors towards a more exophilic and exophagic nature, as well as a shift to zoophilic blood feeding [16,17,18,19,20]. Though the endophilic and endophagic population has been significantly reduced through indoor interventions [21], the behavioral changes in some locations with high net coverage allow these mosquitoes to evade the control tools predominantly used indoors. This leaves mosquitoes with more plastic traits surviving in small numbers that might increase into larger populations with time [22]. This is a major concern for malaria vector control programs that mainly target endophilic, nocturnal, anthropophilic, and endophagic mosquitoes. The potential of zooprophylaxis, which involves diverting mosquitoes to an animal that serves as a dead-end host, in reducing malaria transmission was recognized as early as 1957 and has recently been reported [23,24,25] to help in vector control.

Apart from mosquitoes, cattle are also hosts to other various vectors of human and livestock diseases, such as tsetse flies and ticks. The economic losses caused by arthropod pests as well as arthropod disease vectors in cattle production are estimated to be around USD 1.0–1.2 billion annually [26,27]. This is because vector-infested livestock are stressed by the painful bites and also become sick from transmitted parasite infections. These result in nutritional loss and disease cases that lead to reduced production of milk, meat, and manure [28,29]. For instance, human African trypanosomiasis and African animal trypanosomiasis, transmitted by tsetse flies [30], can have a profound impact on human and livestock productivity, food security, and the financial well-being of farmers in sub-Saharan Africa. On the other hand, ticks transmit a wide range of economically important blood parasites affecting both humans and livestock, such as East Coast fever, babesiosis, anaplasmosis, and various rickettsioses [30,31,32]. In a Tanzanian study, treatment of cattle with a vector control agent was shown to have great potential in vector control, estimated to have increased the daily mortality of tsetse flies by 5–14% [33]. Cost–benefit analysis showed that controlling tsetse flies and ticks by treating 50% of cattle had a higher return than the untreated group in the production of milk, beef, leather, and even drought power [34].

The cattle-targeted approach of exposing vectors to control agents treated on cattle has the potential of controlling host-seeking and blood-feeding vectors [35,36,37]. This emphasizes the need to explore the efficacy of biological control agents and the tools that are beyond one vector and one disease, as well as pose no risk to humans, livestock, plants, and the environment [9]. Apart from being integrated with core malaria control interventions such as insecticide-treated bed nets and indoor residual spraying, zooprophylaxis can also be integrated with zoopotentiation in a biological, cattle-targeted vector control approach [23,24,25]. However, the potential for successful zooprophylaxis relies on host fidelity, and malaria vectors such as *Anopheles arabiensis*, though generally preferring exophilic and zoophilic feeding, and *Anopheles funestus*, which is more endophilic and anthropophilic, clearly show plasticity in host- preference [38,39]. Although a successful blood meal on cattle or other livestock reinforces preference for that host through learning, it is possible that a mosquito may feed on humans in subsequent gonotrophic cycles after feeding on an animal [40]. However, if the learning leads to cattle preference, and if cattle are treated with a vector control agent, the first, and, /or, successive blood feeding leads to more exposure to the agent;, hence, the vectors may die earlier before the next, or, a successive blood feeding and, thereby, reducing disease transmission to humans. In relation to this approach of biorational agent treatment on cattle, incorporation of approaches such as zooprophylaxis and zoopotentiation may have great potential in infecting and killing the vectors upon landing and blood feeding on cattle treated with the vector control agents [35,36,37].

Considering the public health importance of these vectors, especially in low-income countries that rely on keeping livestock for livelihood but lack adequate capacity to manage vectors and disease outbreaks, this study explored the potential of an entomopathogenic fungus in biological arthropod vector management. It involved testing for the efficacy of *Metarhizium anisopliae* against mosquitoes, tsetse flies, and ticks.

Generally, the *M. anisopliae* fungus spores attach to the arthropod on contact, germinate, and differentiate into infection structures, appressoria, and penetration pegs, which penetrate through the cuticle using enzymes and mechanical force to colonize the hemocoel. Following the death of the host, the conidiophores emerge on the cadaver and produce spores [41]. The multiple modes of action in the infection process reduce the chances of the arthropod host developing resistance [42]. Different isolates of *M. anisopliae* have previously been shown to infect and kill different arthropod vectors [43]. For instance, the *M. anisopliae* isolate ICIPE 7 has been found to be effective against different species of ticks, while isolate ICIPE 30 is effective against mosquitoes and tsetse flies [16,44,45,46,47]. Due to its efficacy against ticks [43], ICIPE 7 was commercialized as Mazao TICKOFF^®^ by Real IPM, Kenya [48], for use in cattle spraying as a bio-acaricide. However, ICIPE 30 has not yet been registered for public use. In considering the role of cattle as the blood host for a multitude of human and animal disease vectors, a commercial biorational product might have benefits beyond control of the targeted vectors alone, hence providing an opportunity for integrated vector management.

In this study, we aimed to evaluate the suitability of *M. anisopliae,* isolate ICIPE 7, in simultaneously infecting and killing mosquitoes, tsetse flies, and ticks. This was achieved by (1) comparing the efficacy of the two isolates, ICIPE 7 and ICIPE 30, against mosquitoes; (2) determining the efficacy of ICIPE 7 against tsetse flies and ticks, on the confirmation of its efficacy against mosquitoes; (3) determining mosquito survival rates upon exposure to different ICIPE 7 spore concentrations; (4) assessing the differences in mortality rate of mosquitoes exposed to ICIPE 7 for different time periods; (5) testing the spore attachment upon mosquito exposure to different ICIPE 7 concentrations and also the spore attachment to tsetse flies and *Rhipicephalus* ticks when exposed to the selected ICIPE 7 concentration.

This study aimed at testing the efficacy of the ICIPE 7 formulation based on the isolate being already registered for public use as a tick control product, Mazao TICKOFF^®^, and readily being available for use. This would make it possible to conduct field studies for efficacy tests against the targeted mosquitoes, tsetse flies, and ticks. The product was formulated to be sprayed on cattle, which is our expected target host that exposes the fungal spores to host-seeking vectors. Also, the fungal isolate had the possibility of still being able to infect and kill other arthropod disease vectors, even if at a slower rate or with lesser virulence than that against ticks. If ICIPE 7 formulation had not been found to be effective against mosquitoes, it would have been tested against tsetse flies and ticks. However, since ICIPE 7 infected and killed mosquitoes too, it had to be tested further, even against tsetse flies and ticks, thus leaving out ICIPE 30, which was important in helping us compare the efficacy of ICIPE 7 in relation to its efficacy against mosquitoes. Even though the efficacy of ICIPE 30 against mosquitoes was higher than the efficacy of ICIPE 7, both isolates led to mosquito mortality within the second week from fungal infection; hence, ICIPE 7 is preferred due to its public use approval and its ability to also infect and kill additional arthropod vectors.

## 2. Materials and Methods

### 2.1. Study Site

All experiments were conducted at the International Centre of Insect Physiology and Ecology’s Thomas Odhiambo Campus (*icipe*-TOC), at Mbita, Homa Bay County, Western Kenya. The campus is located along the shores of Lake Victoria (0°26′06.19″ S, 34°12′53.13″ E; altitude 1137 m above sea level). In order to prevent contamination of other research areas and insectary facilities, experiments were set up in an isolated makeshift semi-field structure, with walls made of a shade net fabric covered with straw mats, to prevent overheating. The roof was covered with corrugated iron sheets, while the floor was covered with sand (30 cm deep) which was watered constantly to maintain a relative humidity higher than 70%.

### 2.2. Arthropod Source

Female *An. arabiensis* (Mwea strain) two to three days old were obtained from the mosquito insectary at *icipe*-TOC. Wild riverine tsetse flies, *G. fuscipes*, were collected from the shores of Lake Victoria, within *icipe*-TOC using bi-conical traps. Wild, engorged nymph and adult ticks were hand-picked from cattle held at *icipe*-TOC for animal health research. The ticks were of different species within the *Rhipicephalus* genus, since they are the only ticks that were found and collected from the cattle. These were only identified to genus level and had ICIPE 7 tested against them for efficacy assessment.

### 2.3. Insectary Mosquito Rearing

The insectary mosquitoes received from *icipe*-TOC insectary were reared within separate larval and adult rooms, following developed and approved SOPs. The average temperature in the larval and adult rooms was 24–32 °C and 24–30 °C, respectively. The female mosquitoes were fed on 6% glucose but starved for about 2 h whenever they were to be blood fed. They were blood fed on the arms of consenting adult human volunteers, following ethical requirements, and then the 6% glucose solution was restored. Oviposition cups were set up for mosquito egg laying, and the eggs were released into raw lake water in trays for hatching. The larvae were fed on Go cat food^®^ to pupation, after which the emerging adults were fed on 6% glucose solution.

### 2.4. Metarhizium anisopliae Isolates

Two isolates of *M. anisopliae*, ICIPE 7 and ICIPE 30, were obtained from the Arthropod Pathology Unit of *icipe*-TOC. The viability of spores, assessed as percentage germination, was determined before every experiment, and to ensure maximum performance, only spores with over 85% viability were used [49]. Dry spores were stored in a refrigerator at 4 °C.

### 2.5. Fungus Formulation and Arthropod Exposure

Stock suspension for each isolate was prepared by adding 1 g of spores to 10 mL of 0.1% Triton^®^ X-100 (Sigma-Aldrich, St. Louis, MO, USA). Solutions of the required concentrations were made through serial dilutions of this stock suspension. The suspension was vortexed, and 1 mL was spread on waxy printing paper, using a K-bar hand coating machine [50]. The spore-coated papers were allowed to dry in a shaded space for 1 h and then individually inserted into cylindrical PVC tubes (21 cm long with a diameter of 6.2 cm, giving an internal surface area of 409.1 cm^2^), with the coated side exposed for the landing of the arthropods. The selected ICIPE 7 concentration of 10^9^ spores/mL had a spore concentration of 2.4 × 10^6^ spores/cm^2^ on the exposure paper, which had a surface area of 409.1 cm^2^. The two ends of the PVC tube were covered with mosquito net fixed with rubber bands, to prevent mosquitoes from escaping. As control, an equal amount of 0.1% Triton X-100 was spread on the waxy paper, which was placed in a PVC tube after drying.

The control and treatment PVC tubes were placed on designated working benches for every group (control, ICIPE 7, and ICIPE 30) to avoid cross-contamination. The test arthropods were separately released into the grouped PVC tubes through a hole in the netting, which was afterwards closed with a cotton wool plug. During exposure of mosquitoes and tsetse flies, the tubes were kept vertically upright, whilst for ticks the tubes were kept horizontally for the ticks to freely crawl over the exposure paper. After the set exposure periods for mosquitoes and tsetse flies, the tubes were individually placed at the opening of the 15 cm × 15 cm × 15 cm holding cages. This was to allow the arthropods to fly or crawl out of the tube and into the cage. The tube was tapped a bit to cause some disturbance, making the arthropods fly or crawl swiftly into the cages. However, for the ticks, the exposure paper was slowly pulled out of the tube and unrolled to rest flat on the table. The ticks were immediately picked one after the other using disinfected forceps and placed in individual Eppendorf tubes. The used PVC tubes were disinfected and washed separately for every group.

In the individual cages, after exposure to fungus, mosquitoes were supplied with 6% glucose solution, while tsetse flies were fed on cattle blood once a day by placing their cages on cattle skin where the flies probed through the cage netting for blood feeding. The ticks were provided with fresh cotton swabs soaked in clean water and placed at the top of the Eppendorf tubes daily. The arthropods were monitored daily for mortality, until all individuals in a treatment group died. The cadavers of the dead arthropods were surface sterilized by dipping in absolute ethanol and placed on moist filter paper in Petri dishes. They were then incubated at 27 °C for 3–10 days to allow for mycosis, which was checked daily. All the experiments were conducted in three subsequent rounds, each with 3 replicates running at the same time.

### 2.6. Bioassays to Compare the Efficacy of Isolates ICIPE 7 and ICIPE 30 against Female An. arabiensis

The stock suspensions of ICIPE 7 and ICIPE 30 were diluted to a concentration of 10^9^ spores/mL, which has been published as effective against *An. arabiensis* for ICIPE 30 [45]. The mosquito exposure was conducted as described above, with each replicate consisting of 30 2–3-day-old female mosquitoes exposed for 10 min and observed for daily mortality.

### 2.7. Bioassays to Assess the Efficacy of ICIPE 7 against G. fuscipes and Rhipicephalus Ticks

In each replicate of every round of experiments, 20 tsetse flies, 10 tick nymphs, and 10 adult ticks were separately exposed to the 10^9^ spores/mL concentration of ICIPE 7 for 10 min. After exposure, the tsetse flies were transferred to the tsetse fly holding cages, while ticks were transferred to individual 1.5 mL Eppendorf tubes, and then observed for daily mortality.

### 2.8. Bioassays to Establish Concentration-Based Efficacy of ICIPE 7 against An. arabiensis

The lethality of varying concentrations of ICIPE 7 on insectary-reared *An. arabiensis* was tested by diluting the stock suspension to concentrations of 10^6^, 10^8^, 10^9^, and 10^10^ spores/mL. This was to help determine any differences in the efficacy of the different spore dosages, to guide the formulation of an effective concentration to be developed for field trial use. Exposure papers were coated as described earlier, starting with the lowest concentration of 10^6^ spores/mL, and with the mosquitoes being exposed for 10 min. Each replicate consisted of 30 2–3-day-old mosquitoes.

### 2.9. Bioassays to Assess Exposure Period Influence on Time to Death for An. arabiensis

To test if a reduced exposure period to ICIPE 7 would impact the mortality rate of mosquitoes, they were exposed to the selected dosage of 10^9^ spores/mL ICIPE 7 formulation. This concentration was identified from previous experiments, and the mosquitoes were exposed for 1, 3, 5, or 10 min, as described above. Each replicate consisted of 30 2–3-day-old female mosquitoes.

### 2.10. Quantification of Spores Attached to the Arthropods on Exposure

For every replicate, 10 mosquitoes were exposed to different ICIPE 7 concentrations (10^6^, 10^7^, 10^8^, and 10^9^ spores/mL) for 10 min. For the tsetse flies and ticks, each replicate consisted of 5 mixed-age individuals that were, however, exposed to only the selected 10^9^ spores/mL concentration for 10 min. After exposure, the arthropods were transferred to holding cages, or Eppendorf tubes for ticks, and frozen to death. Using disinfected forceps, individual arthropods were picked up and placed in 1.5 mL Eppendorf tubes. Then, 100 µL of Triton X-100 liquid medium solution was added into each Eppendorf tube and vortexed to dislodge spores from the arthropods. Afterwards, 20 µL of the solution was immediately pipetted onto a hemocytometer for spore visualization and counting under a compound microscope (×100 magnification) [45,49], after 5–10 min of allowing the spores in the solution to settle.

### 2.11. Data Analysis

Lethal median time to death of the arthropods and pairwise comparison after exposure to different test regimens were determined by Kaplan–Meier (KM) analysis. If more than half of the arthropods in the control groups remained alive by the time all treatment arthropods had died, the LT_50_ values for the control could not be generated. For experiments that had control in every group, the survival data for the controls were compared and pooled together if there was no significant difference between them. Hazard ratios (HRs) were calculated using Cox regression model for all treatments, with the control group as the baseline, except for the exposure period influence test, where 10 min was set as the baseline. The round and replicate were first added as covariates in the model and removed when not significant, but they were included if they resulted in any significant differences. The probability of dying was calculated by the following equation:

*P* = *HR*/(1 + *HR*)

To compare the number of spores attached to exposed arthropods, Kolmogorov–Smirnov tests were used to test the normality of data. If the data were not parametric, Kruskal–Wallis tests for independent samples were used to compare the medians of different treatments. Generalized linear models were used to compare the number of spores attached (odds ratio, OR) to exposed arthropods. Odds ratio was changed to percentage with the following equation: Percentage (%) = (OR − 1)100. All the analysis was performed using IBM SPSS statistics version 27 (SPSS Inc., Chicago, IL, USA).

## 3. Results

### 3.1. Comparative Efficacy of ICIPE 7 and ICIPE 30 against An. arabiensis

**Figure 1:**
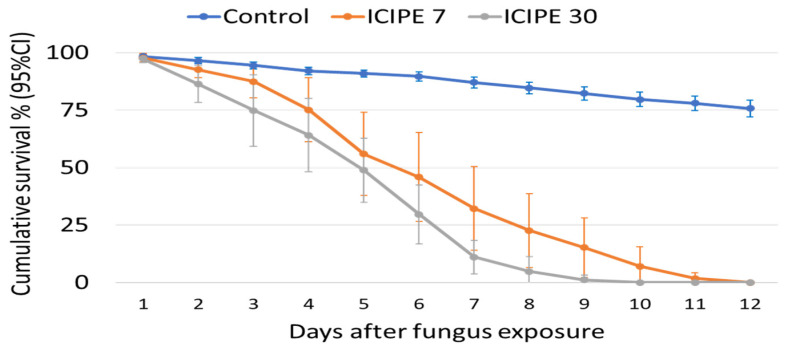
Cumulative survival percentage (95% CI) of *Anopheles arabiensis* adults over time (days) after 10 min of exposure to *Metarhizium anisopliae*, isolates ICIPE 7 and ICIPE 30, at a concentration of 10^9^ spores/mL.

In total, 270 *An. arabiensis* mosquitoes were exposed to each fungus isolate. Both ICIPE 7 and ICIPE 30 infected and killed exposed *An. arabiensis* mosquitoes, with ICIPE 30 generally killing the mosquitoes slightly faster than ICIPE 7. After 10 min of exposure, the LT_50_ (95% CI) of mosquitoes exposed to ICIPE 7 was 6 (5.5–6.5) days, while that of those exposed to ICIPE 30 was 5 (4.6–5.3) days. Pairwise comparison indicated a significant difference in survival (KM analysis; χ^2^ = 40, *p* < 0.001) of mosquitoes after exposure to ICIPE 7 or ICIPE 30, with the mosquitoes exposed to ICIPE 30 (HR (95% CI), 16.6 (13–21), *p* < 0.001) more likely to die (*p* = 0.94 vs. *p* = 0.91) than those exposed to ICIPE 7 (HR (95% CI), 10.9 (8.7–13.7), *p* < 0.001), when compared to control mosquitoes at any time point (Figure 1). The LT_50_ for the control groups could not be generated, as more than half of the exposed mosquitoes were still alive by the time all treatment mosquitoes had died.

### 3.2. Efficacy of ICIPE 7 against wild G. fuscipes and Rhipicephalus Ticks

**Figure 2:**
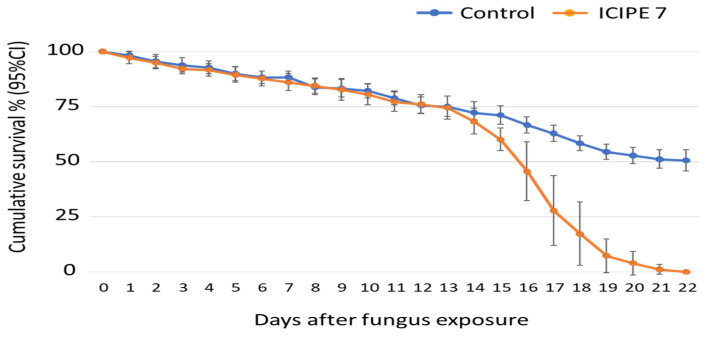
Cumulative survival percentage (95% CI) of *Glossina fuscipes* adults over time (days), after 10 min of exposure to *Metarhizium anisopliae*, isolate ICIPE 7, at a concentration of 10^9^ spores/mL.

In total, 180 tsetse flies were exposed to 10^9^ spores/mL of ICIPE 7-treated exposure paper for 10 min. The exposure significantly reduced the survival of wild-caught *G. fuscipes* of unknown age (Figure 2). While the LT_50_ was 16 (15.4–16.5) days for the ICIPE 7 group, the control flies only reached 50% mortality by day 22, at which time point the experiment was terminated, since all treatment flies had died. Overall comparison of the survival curve shows that the flies exposed to ICIPE 7 were more likely to die (*p* = 0.76) compared to the control at any time point (HR (95% CI), 3.3 (2.5–4.3), *p* < 0.001).

**Figure 3:**
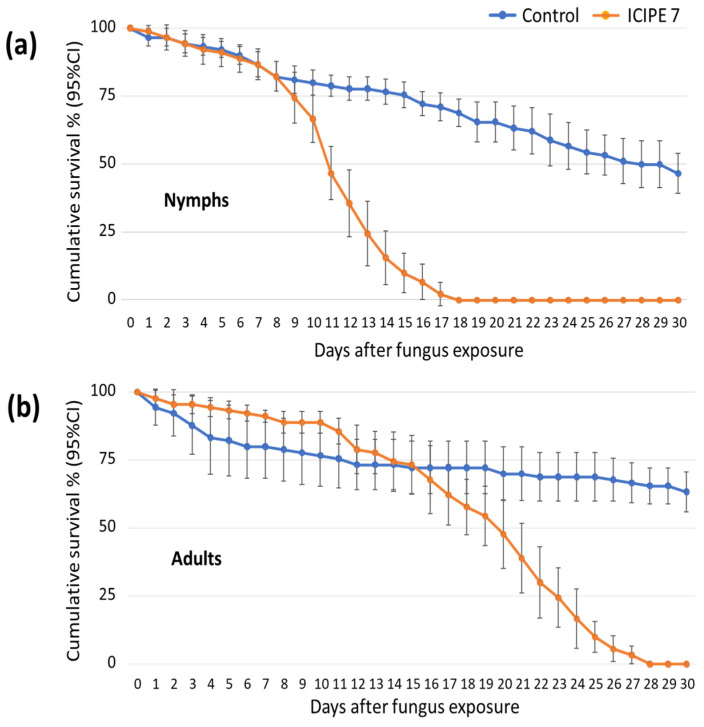
Cumulative survival percentage (95% CI) of (**a**) nymphs and (**b**) adult *Rhipicephalus* ticks over time (days), after 10 min of exposure to *Metarhizium anisopliae*, isolate ICIPE 7, at a concentration of 10^9^ spores/mL.

In total, 90 nymphs and 90 adult *Rhipicephalus* ticks were exposed to 10^9^ spores/mL of ICIPE 7-treated exposure paper. They were susceptible to fungal infection and had significantly reduced survival (Figure 3). The LT_50_ was 11 days (10.3–11.7 days) for the nymphs (Figure 3a) and 20 days (18.4–22 days) for the adults (Figure 3b). Overall comparison of survival showed that both nymphs (HR (95% CI), 7 (4.4–11), *p* < 0.001) and adults (HR (95% CI), 7 (4.4–11), *p* < 0.001) were equally likely to die (*P* = 0.86) when compared with their respective controls.

### 3.3. Concentration-Dependent Efficacy of Dosages around 10^9^ Spores/mL

**Figure 4:**
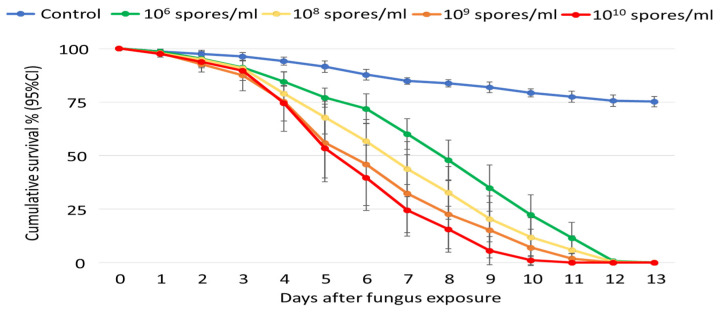
Cumulative survival percentage (95% CI) of *Anopheles arabiensis* adults over time (days), after 10 min of exposure to *Metarhizium anisopliae*, isolate ICIPE 7, at concentrations of 10^6^, 10^8^, 10^9^, and 10^10^ spores/mL.

In total, 270 mosquitoes were exposed to four concentrations: 10^6^ spores/mL, 10^8^ spores/mL, 10^9^ spores/mL, and 10^10^ spores/mL. Overall, there was a concentration-dependent effect with faster mortality associated with higher concentrations (Figure 4), and the likelihood of dying (*P*) ranged from 0.89 for 10^6^ spores/mL to 0.94 for the 10^10^ spores/mL (Table 1).

The LT_50_ decreased with increases in concentration, being 8 days and 7 days for concentrations of 10^6^ spores/mL and 10^8^ spores/mL, respectively, while being 6 days for both 10^9^ spores/mL and 10^10^ spores/mL (Table 1). However, the pairwise comparison did show a significant increase in the overall mortality (KM analysis; χ^2^ = 8.2, *p* = 0.004). With the control as the baseline, hazard ratios also increased with the increase in fungus concentration.

### 3.4. The Exposure Period Influence on Time to Death for An. arabiensis

**Figure 5:**
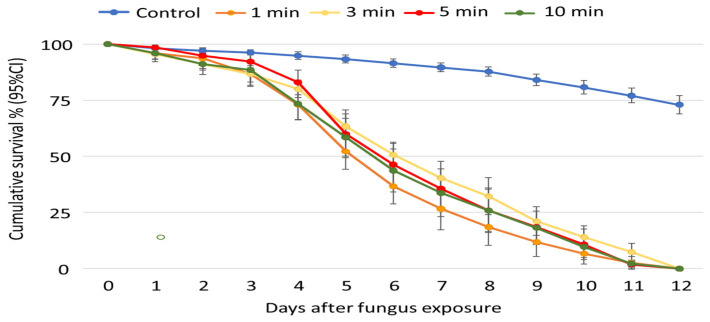
Cumulative survival percentage (95% CI) of *Anopheles arabiensis* adults over time (days), after exposure to 10^9^ spores/mL of *Metarhizium anisopliae*, isolate ICIPE 7, for 1, 3, 5, and 10 min.

Similar to the bioassays above, a total of 270 mosquitoes were exposed to 10^9^ spores/mL of ICIPE 7 for 1, 3, 5, and 10 min, respectively. There was no difference in the survival of the control groups (HR (95% CI), 0.97 (0.8–1.1), *p* = 0.6) for the exposure periods; hence, they were pooled for further analysis (n = 1080). Overall, longer exposure periods did not have an additional impact on mortality (Figure 5).

Apart from the LT_50_ of 7 days for mosquitoes exposed for 3 min, the LT_50_ remained the same for all exposure periods (Table 2). Pairwise comparison also showed no significant increase in the overall mortality following exposure to fungus spores for 3, 5, and 10 min. With the 10 min exposure period as the baseline, the hazard ratio was only significant for control mosquitoes, indicating a low likelihood (*p* = 0.09) of dying (Table 1). The HRs of mosquitoes exposed for 1, 3, and 5 min were similar to that of 10 min of exposure to ICIPE 7 formulation.

### 3.5. Concentration-Dependent Spore Attachment to Mosquitoes upon Exposure to ICIPE 7

**Figure 6:**
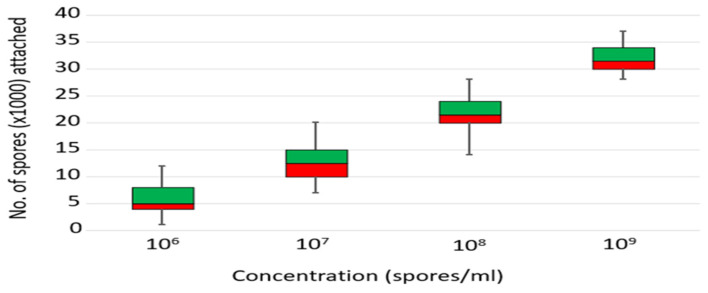
Box plot of number of ICIPE 7 spores attached to *Anopheles arabiensis* adults, following exposure to 10^6^, 10^7^, 10^8^, and 10^9^ spores/mL. Within each box, horizontal line denotes median values, boxes extend from the 25th to the 75th percentile, and the error bars indicate the range. Pairwise comparison shows significant difference (*p* < 0.001) in spores attached amongst all the concentrations.

A significantly higher number of spores attached to *An. arabiensis* mosquitoes when exposed to increasing concentrations of ICIPE 7 for 10 min (Kruskal–Wallis test; χ^2^ = 327, df = 3, *p* < 0.001) (Figure 6). Pairwise comparison showed a significant difference (*p* < 0.001) amongst all the concentrations. The mosquitoes exposed to 10^6^ spores/mL had a median of 5 × 10^3^ spores/mosquito. Those exposed to 10^7^ spores/mL had 120% (OR 95%(CI), 2.15 (2.14–2.15), *p* < 0.001) more spores attached, compared to exposure to 10^6^ spores/mL, while those exposed to 10^8^ spores/mL had 270% (OR 95%(CI), 3.65 (3.64–3.77), *p* < 0.001) more spores, and those exposed to 10^9^ spores/mL had 436% (OR 95%(CI), 5.36 (5.3–5.37), *p* < 0.001) more spores attached.

### 3.6. Spores Attached to G. fuscipes and Rhipicephalus Ticks upon Exposure to the Fungus

**Figure 7:**
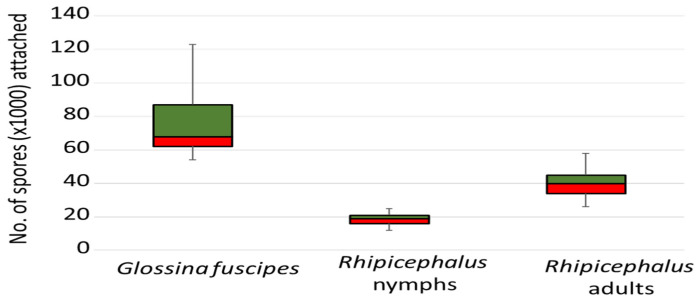
Box plot of the number of ICIPE 7 spores attached to *Glossina fuscipes*, as well as nymph and adult *Rhipicephalus* ticks following exposure to 10^9^ spores/mL for 10 min. Within each box, horizontal line denotes median values, boxes extend from the 25th to the 75th percentile, and the error bars indicate the range.

Pairwise comparison showed a significant difference (*p* < 0.001) in the number of spores attached to *G. fuscipes* and nymphal as well as adult *Rhipicephalus* ticks (Figure 7). *G. fuscipes* had the highest average number of spores attached (74,544 (S.D. 17,530) spores/fly), followed by adult ticks (40,422 (S.D. 7614) spores per tick) and then the nymphal ticks (18,911 (S.D. 3566) spores per tick). Compared to adult ticks, *G. fuscipes* had 84% (OR 95%(CI), 1.84 (1.841–1.847), *p* < 0.001) more spores attached, while nymphal ticks had 53% (OR 95%(CI), 0.468 (0.467–0.469), *p* < 0.001) less spores attached.

## 4. Discussion

In this study, we showed that both isolates of *M. anisopliae,* isolates ICIPE 7 and ICIPE 30, infect and kill *An. arabiensis* mosquitoes and hence may be used in their control. ICIPE 7 has previously been shown to infect and kill species of ticks [44] and has more recently been confirmed to infect and kill fall armyworm larvae [51]. Our findings further illustrate the potential of this isolate in the management of multiple vectors of medical and veterinary importance.

ICIPE 7 killed all exposed insectary *An. arabiensis* mosquitoes, albeit at a slightly slower rate than ICIPE 30, irrespective of the exposure period. This is similar to previous studies that showed high ICIPE 30 virulence against mosquitoes [16,45], likely a result of selecting the strain against them. However, our results suggest that ICIPE 7-based products applied on cattle for tick control might well impact the survival of mosquitoes seeking cattle as a blood host.

Biological control tools are slow-acting due to their multiple biological modes of action, when compared to insecticides [42]. The slow action is acceptable if the isolates still have the potential to achieve reductions in disease transmission, comparable to those achieved with instant-kill insecticides [52]. It takes 10–18 days for *Plasmodium* sporozoites to develop and migrate to mosquito salivary glands [53], from which they are injected into a human host during blood feeding. Our results show that while most *Plasmodium*-infected mosquitoes will die of ICIPE 7 infection before transmitting the parasites, 0–20% of the mosquitoes can contribute to *Plasmodium* transmission before their death [54,55]. However, previous work showed that *M. anisopliae* infection also reduces host-seeking and blood-feeding propensity, which might contribute to lower risk of *Plasmodium* transmission in surviving mosquitoes [56].

Our results further suggest that even one minute exposure to ICIPE 7-treated substrate is sufficient to reduce mosquito survival significantly. Our results agree with Scholte et al. [57], who found no effect of exposure period on *An. gambiae s.s.* survival upon using ICIPE 30. In contrast, another study showed a significant difference in mortality of *An. gambiae s.s.* after 15 min and 30 min of exposure to ICIPE 30 [45]. The similarities or differences in the mortality rate might have resulted from the different mosquito colonies or the exposure method used in the involved studies. These results are encouraging, as in nature mosquitoes will contact the treated host for a few minutes only. Visualization of *An. gambiae* feeding on mice in the laboratory showed that, on average, 54.6 ± 95 s are spent on probing and 110 ± 17 s on blood feeding [58]. Although this may vary depending on if the mosquitoes are infected with *Plasmodium* or under natural settings, this indicates that the time spent on blood feeding would be sufficient to receive a lethal inoculum of fungus.

The 10^9^ spores/mL ICIPE 7 formulation also kills wild tsetse flies, as well as wild tick nymphs and adults. While there are studies that tested the efficacy of isolate ICIPE 30 against tsetse flies [46,47], ours is the first study to report the efficacy of ICIPE 7 against them. Conversely, the fungus has been tested extensively against ticks. For instance, a previous study [59] reported 100% mortality in *Rhipicephalus* spp. and *Amblyoma* spp. from exposure to *M. anisopliae,* in all their developmental stages. Also, another laboratory study [60] confirmed the susceptibility of *Rhipicephalus decoloratus* larvae to *M. anisopliae,* isolate ICIPE 7, with a mortality of up to 100%, having an LT_50_ of 2.6–4.2 days. In addition to this, the tick survival in this study had a slight variation from what has been previously reported. The findings by Kirkland et al. [61] indicated mortality of exposed *Rhipicephalus* nymphs to have been >90% by day 21, while adults were at >70% by day 28 post-treatment, which slightly varies from our findings of 100% nymphal mortality by day 18 and adult mortality by day 28. A few possible explanations for this divergence could be the use of wild ticks vs. insectary-reared ticks, age-unmatched and age-matched samples, differences in exposure methods (exposure to treated paper vs. submerging and spraying), or use of different *M. anisopliae* isolates. However, as mentioned above, the key goal is to reduce disease transmission through vector control, and a variation in mortality rate only compromises the control agent if that mortality is not achieved at a significant level.

Tsetse flies transmit trypanosomes, feed almost daily, and live for about 4 months. With the fungal treatment efficacy against tsetse flies, as based on the bioassays, the 50% reduction in population only after 16 days following fungus exposure will initially not have much impact on disease transmission. However, the population reduction, combined with fly to fly spore transfer by fungus-infected flies [62], low fecundity rate of tsetse flies (one pupa/10 days), and the non-lethal effects of fungus infection, such as reduced feeding and fertility, may contribute to reductions in vector population as well as disease transmission [63]. Ticks, on the other hand, transmit pathogens as they stay attached to the host while feeding for days and live for up to 3 years [64]. In this case, a more than 50% reduction in tick population by the fungal agent by day 20 is expected to have an earlier impact on disease transmission.

All the tested ICIPE 7 concentrations of 10^6^, 10^8^, 10^9^, and 10^10^ spores/mL were effective against *An. arabiensis,* with a dose-dependent effect on mortality. This is similar to studies [45,65] that indicated dose-dependent mortality of *An. gambiae* when exposed to *M. anisopliae*, though with isolate ICIPE 30. An important point is that even the lowest dose of 10^6^ spores/mL caused >75% mortality at day 10 in the laboratory bioassays. However, in extreme cases of high spore loss, or spore viability loss, formulations with slightly higher spore concentrations are preferred, even with up to 10^10^ spores/mL, which is not cost-effective due to the high amounts of spores required for any field application.

By quantifying the spore density on exposed arthropods, we aimed to confirm if increasing the number of spores was consistent with an increase in mortality due to mycosis. In addition, this was a validation that exposure to a fungus-treated surface is a suitable delivery method for the three very different arthropods. Treatment mosquitoes had a significant number of spores attached compared to the control, which increased with the spore concentration. At the selected dose of 10^9^ spores/mL, the spore attachment in the order of lowest to highest spore numbers was on tick nymphs, followed by mosquitoes, adult ticks, and tsetse flies. This is consistent with the arthropod body size and the body structure. It therefore shows that a fungus-treated surface is an effective fungal formulation delivery method. This supports the idea of targeting multiple arthropods by spraying the fungal formulation on cattle, assuming that the fur on the cattle’s skin does not affect the availability of spores. This result is similar to the findings by Scholte et al. [66], who recorded spores attached to *An. gambiae s.s* females when exposed to *M. anisopliae*, ICIPE 30, using exposure papers and added that spores were even transferable to males during mating. However, this would really work in cases of females becoming exposed immediately before mating, greatly increasing the chances of fungal infection and hence a mosquito population reduction. The study by Maniania [47] showed that tsetse flies had 10^5^–10^6^ spores/mL attached when exposed to ICIPE 30 for less than 60 s, in a designed 1 L water bottle contamination device. This concentration is higher than the 10^4^ spores/mL attached after 10 min of exposure in this study, which is probably due to the delivery method, as dry spore powder was used in their exposure, and it easily gets attached when contacted. This is unlikely for our case of spores being formulated in a surfactant and coated on a paper, indicating that the difference in the number of spores attached is dependent on the delivery method used [49].

The attachment of spores to exposed ticks in this study was 1.0–4.0 (×10^4^) spores/individual nymph or adult, which slightly differs from the findings of Ment et al. [67] that recorded 2.5 × 10^4^–2.6 × 10^5^ spores/adult tick when exposed to 10^6^–10^8^ spores/mL of *M. anisopliae var. anisopliae* strain 7. This is likely a result of their different exposure method, which involved dipping individual ticks into the fungal suspension, resulting in a higher number of spores attached.

One limitation of our experiments with tsetse flies and ticks is the fact that we could not age-standardize the organisms used, given that we had to collect them from the wild, which may have affected their survival. However, this equally applies to control and treatment groups; hence, our overall conclusion is not expected to greatly differ with experiments of age-standardized arthropods.

## 5. Conclusions

This study shows that *M. anisopliae*, isolate ICIPE 7, infects and kills *An*. *arabiensis*, *G. fuscipes*, and both nymphs and adults of *Rhipicephalus* spp. ticks, the vectors of major human and livestock diseases. The observed mortality results from mycosis, due to ICIPE 7 spore attachment and infection of the arthropods upon exposure to ICIPE 7-treated surfaces. However, due to the slow death of the exposed vectors, these laboratory findings need further validation in the field to confirm the ability of ICIPE 7 in reducing the wild populations of multiple disease vectors in greatly affected sub-Saharan regions.

## Figures and Tables

**Table 1:** Pairwise comparison, lethal median time (LT_50_ 95% CI) to death (in days), and hazard ratio (HR 95% CI) of *Anopheles arabiensis* adults after 10 min of exposure to *Metarhizium anisopliae*, isolate ICIPE 7, at concentrations of 10^6^, 10^8^, 10^9^, and 10^10^ spores/mL.

Concentration (Spores/mL)	Pairwise Comparison (*p*-Value) *				LT_50_ (95% CI) * in Days	HR (95% CI) ^!^
	Control	10^6^	10^8^	10^9^		
10^6^	<0.001				8 (7.5–8.5)	7.9 (6.4–9.8) ^a^
10^8^	<0.001	<0.001			7 (6.5–7.4)	10.3 (8.3–12.3) ^a^
10^9^	<0.001	<0.001	0.002		6 (5.5–6.5)	12.8 (10.2–15.8) ^a^
10^10^	<0.001	<0.001	<0.001	0.004	6 (5.6–6.3)	15.4 (12.3–19.3) ^a^

* Pairwise comparison and LT_50_ calculated with Kaplan–Meier analysis. ^!^ HRs calculated with control as baseline (Cox regression). Superscript “a” indicates significant HR.

**Table 2:** Pairwise comparison, lethal median time (LT_50_ 95% CI) to death (in days), and hazard ratio (HR 95% CI) of *Anopheles arabiensis* adults after exposure to 10^9^ spores/mL of *Metarhizium anisopliae*, isolate ICIPE 7, for 1, 3, 5, and 10 min.

Exposure Time (min)	Pairwise Comparison (*p*-Value) *				LT_50_ (95% CI) * in Days	HR (95% CI) ^!^
	1	3	5	10		
Control	<0.001	<0.001	<0.001	<0.001	na	0.1 (0.85–0.12) ^a^
1	-	0.004	0.12	0.08	6 (5.7–6.3)	1.1 (0.9–1.3)
3	-	-	0.7	0.3	7 (6.6–7.4)	0.9 (0.8–1.1)
5	-	-	-	0.47	6 (5.5–6.5)	0.9 (0.8–1.1)
10	-	-	-	-	6 (5.6–6.4)	na

* Pairwise comparison and LT_50_ calculated with Kaplan–Meier analysis. ^!^ HRs calculated with the 10 min exposure time as baseline (Cox regression). Superscript “a” indicates significant HR.

## Data Availability

The data leading to the conclusions of this work are presented within this article. All data analyzed are available and will be provided by the corresponding author upon reasonable request.
